# Supplementary material for: Visitors’ support of ocean protection in a low-use marine protected area: Gray’s reef national marine sanctuary
Source: PLoS One. 2025 Nov 7;20(11):e0336140. doi: 10.1371/journal.pone.0336140 (PMC12594416; doi:10.1371/journal.pone.0336140)
Supplement: Attachment — Submitted filename: My comments.docx [file pone.0336140.s002.docx]

General comments: The manuscript addresses a timely and relevant topic concerning public support for ocean protection in a marine protected area (MPA), specifically Gray's Reef National Marine Sanctuary (GRNMS). The study is well-structured and aligns with conservation priorities, several areas require improvement. But I have itemized some important comments below that the authors should attend to; in areas where the request was already included but I didn’t catch it, please point those out.

Specific comments:

1. The title could be more precise by specifying the region studied (e.g. “....at Gray’s Reef National Marine Sanctuary, Georgia”), especially since the study emphasizes spatial variation in perceptions.
2. The abstract is overly descriptive and lacks specificity in methodology and key statistical results. For example, the phrase “relatively high support” should be backed with exact figures or mean scores. It would benefit from including the statistical tools used (e.g. multi-model inference, regression analysis) and one or two concrete numerical results I think.
3. The introduction overuses general claims without proper citations or logical flow in parts. For instance, the second sentence, “Thus, the connections people have to the ocean, individually and as a society, are numerous,” sounds generic to me. It would be stronger if the authors cited empirical studies illustrating these connections. Similarly, while the paradox of tourism is discussed, the introduction does not clearly articulate the unique research gap this study fills, especially in comparison with other studies of U.S. marine sanctuaries.
4. The research questions (lines 254, 264, and 277) are buried in the methods. These should be explicitly stated at the end of the introduction to guide the reader. Their current placement dilutes their impact.
5. The paragraph beginning “Understanding environmental values…..” (lines 134) repeats ideas already introduced in 2.1 without contributing new synthesis. This could be removed or integrated more succinctly.
6. The manuscript states that “users of GRNMS are defined as the respondents that reported a visit within 2019” (line 202). However, the initial pool was selected based on fishing license databases. Could this lead to sampling bias, as it excludes non-fishing users like divers or non-consumptive recreationists?
7. The Dillman web method is mentioned, but the authors do not explain how their adjustments were tailored to the study’s context. For example, was any pilot testing done to validate the cultural or regional appropriateness of the questions? Were reminders standardized across all rounds?
8. The sample profile suggests major demographic skewness. The sample is overwhelmingly male (85%) and white (95%). This limitation should be addressed more explicitly in both the discussion and limitations sections, as it strongly impacts the generalizability of the findings.
9. The survey measures section refers to seven sections and 48 questions, yet only those relevant to “support of protection” and “willingness to act” are discussed in the analysis. What happened to the rest? A flowchart or schematic summarizing which items contributed to which indices would improve transparency.
10. It is problematic that only one regression model (M5) was statistically significant, and that adjusted R² values for the other two models were low (M10 = 0.093; M15 = -0.082). I think these weak models should be discussed with more caution, especially given that conclusions are drawn from them. Relying heavily on non-significant findings can mislead the interpretation of the drivers of willingness to act.
11. Table 1 is misleading in presentation. It states that “N = 39” for age but later says total respondents were 99. This discrepancy should be clarified. I don’t get it there, were only 39 respondents included in the socio-demographic analysis?
12. Table 3 uses a 5-point scale but combines responses in a way that may obscure interpretation. For example, reporting means and medians for actions like recycling and donating do not capture the actual behavioral intention variability. Boxplots or histograms would be more informative.
13. The discussion overstates the results in several areas. It claims “relatively high support of protection,” but 24% of respondents expressed no or low support, which is not negligible. Similarly, it says respondents “would do some,” yet the overall willingness to act was modest and skewed away from high-effort behaviors (e.g.. donations, volunteering).
14. The statement that ‘place of residency showed some evidence of a relationship to support” (line 421, ) is based on a p-value of 0.066, which is not statistically significant. So the language should be more cautious here and avoid overstating borderline results.
15. The idea of stewardship is discussed without clearly defining it or showing how it was operationalized in this study. If stewardship is a central theme, the authors should show how survey responses reflect stewardship principles (e.g., personal responsibility, conservation ethics).
16. The conclusion restates the entire paper without providing strong synthesis or actionable insights. It could be shortened and focused more on practical implications for marine sanctuary managers and future survey development.
17. The limitations section is quite strong but appears too late in the manuscript. It might be more impactful if merged with the discussion or presented earlier. The issue of relying solely on anglers for sampling should be emphasized more clearly, as it systematically excludes non-consumptive users. Maybe put it before the conclusion.
