# Supplementary material for: Visitors’ support of ocean protection in a low-use marine protected area: Gray’s reef national marine sanctuary
Source: PLoS One. 2025 Nov 7;20(11):e0336140. doi: 10.1371/journal.pone.0336140 (PMC12594416; doi:10.1371/journal.pone.0336140)
Supplement: Attachment — Submitted filename: Response to Reviewers 9.28.docx [file pone.0336140.s005.docx]

Dear Dr. Inácio,

We thank you, as well as the both reviewers, for your insightful comments on our manuscript (PONE-D-25-19622R1). Please find a point-by-point explanation of how each comment was addressed below. We believe the revisions made have strengthened the manuscript, and that it is now suitable for publication in PLoS ONE.

Editor Comments:

- Please check for typos, extra spaces, and lack of full stops (e.g., L39) in the manuscript

**Response: The manuscript has been thoroughly checked for the punctuation errors noted, and these have been corrected throughout. Additionally, minor wording changes were made throughout to improve the readability of the manuscript.**

- I agree with the reviewer, there is a need for an introductory part in the abstract.

**Response: The following text has been added to the abstract to address the need for additional introductory material: “Marine protected areas (MPAs) are important tools for conserving biodiversity and supporting healthy ocean ecosystems and economies. However, the effectiveness of MPAs is dependent upon the support of those who use and are affected by them. Understanding how recreational users relate to protected areas can provide key insights into long-term support for conservation initiatives.”**

- Chapter 2 is really a literature review, or more like a background.

**Response: This chapter title was changed to “Background” to better align with the content presented.**

- Several sub-chapters are not numbered (e.g., L208) after 3.2 or Model Creation after 3.4

**Response: All sub-chapters have now been numbered and are sequential throughout the manuscript.**

- Line 221: from here is a description of the sample profile. Why here?

Response: This section has been restructured to ensure the relevant text now falls under the Sample Profile heading.

- Line 235: why only 39 respondents

Response: The number varies from 38—40 depending on the variable, clarified in text.

- In Table 1, what is the Latino or Latino and then No? Makes little sense to me.

Response: This was corrected to include “Latino or Latina.”

- Also, in Table 1, why are there 40 valid answers for Education when it was said that 39 was the sample size?

Response: The number varies from 38—40 depending on the variable, clarified in text.

- Finally, the conclusions should be integrated throughout the discussion. Conclusions are meant to give an overview of the results obtained and lay the foundations for future research.

**Response: The separate Limitations and Conclusions sections have been removed, and the content within those sections has been edited and reorganized into a single, cohesive Discussion section.**

Reviewer #1 Comments:

I believe the abstract section still needs some focused refinement. At the moment, it jumps directly into stating the objective without first offering a clear introductory context or problem statement. Starting with a concise and compelling sentence that frames the broader issue would help orient the reader before moving into the purpose of the work.

Additionally, I noticed a few typos, such as "us" instead of "use," as well as some long or inconsistent sentences that could be rephrased for clarity. The strength of a good abstract lies in its ability to deliver a powerful, succinct summary that immediately captures the interest of the target audience. In its current form, I think the abstract doesn’t quite achieve that yet, but with a few revisions particularly in structure and language, it can be significantly improved.

**Response: As noted above, the abstract has been refined to include clear introductory text that better introduces the problem explored in this manuscript. Additionally, minor wording changes were made throughout to improve the readability of the manuscript.**

Sincerely,

Lead author, on behalf of all authors
