# Supplementary material for: Visitors’ support of ocean protection in a low-use marine protected area: Gray’s reef national marine sanctuary
Source: PLoS One. 2025 Nov 7;20(11):e0336140. doi: 10.1371/journal.pone.0336140 (PMC12594416; doi:10.1371/journal.pone.0336140)
Supplement: Attachment — Submitted filename: Response to Reviewers 10.16.25.docx [file pone.0336140.s006.docx]

Response to Academic Editor’s Comments 10/16/25

**ACADEMIC EDITOR:**

**Dear authors, thank you for addressing all the queries.**

**However, before accepting the manuscript, I would like to clarify again the issue of the 39, 40 and 38 respondents. The authors responded that this was explained in the text. However, I do not find this explanation. Could you please explain why the analysis was conducted for only 39 respondents, and in the tables, the numbers of respondents for some categories are listed as 38 and 40? Please clarify this, since this has been an issue in previous versions as well. This is intended to clarify the matter for the manuscript's readers. Please address this.**

The sample size used in all analyses was N=99. The reference to “39, 40, 38 respondents” referred to the total number of respondents who answered an individual survey question in the demographics section. It is not necessary to report each of these totals in the text, so we removed the following sentences to avoid confusion. We also modified Table 1 into a more concise format that is typical for presenting demographic characteristics.

·        Line 228-229: “The socio-demographic analysis included only 39 respondents.”

·        Line 331-333: “Socio-demographic data provided by the state of Georgia further reduced this number to 38—40, depending on the variable.”

**Additionally, consider keeping limitations as a separate sub-chapter within the discussion. And if the authors do not want to include a conclusion, then at least a sub-chapter as concluding remarks.**

Thank you, we added two sub-sections (Limitations and Conclusions).
